# An Earlier First Meal Timing Associates with Weight Loss Effectiveness in A 12-Week Weight Loss Support Program

**DOI:** 10.3390/nu14020249

**Published:** 2022-01-07

**Authors:** Mana Hatanaka, Yoichi Hatamoto, Eri Tajiri, Naoyuki Matsumoto, Shigeho Tanaka, Eiichi Yoshimura

**Affiliations:** 1 Department of Nutrition and Metabolism, National Institute of Health and Nutrition, National Institutes of Biomedical Innovation, Health and Nutrition, 1-23-1 Toyama, Shinjuku-ku, Tokyo 162-8636, Japan; hatanaka@nibiohn.go.jp (M.H.); tanaka.shigeho@eiyo.ac.jp (S.T.); 2 Graduate School of Environmental & Symbiotic Sciences, Prefectural University of Kumamoto, 3-1-100 Tsukide, Higashi-ku, Kumamoto 862-8502, Japan; g1975002@pu-kumamoto.ac.jp; 3 Faculty of Environmental & Symbiotic Sciences, Prefectural University of Kumamoto, 3-1-100 Tsukide, Higashi-ku, Kumamoto 862-8502, Japan; nao-st205@pu-kumamoto.ac.jp; 4 Faculty of Nutrition, Kagawa Nutrition University, 3-9-21 Chiyoda, Sakado City 350-0288, Japan

**Keywords:** meal timing, weight loss support program, body weight

## Abstract

Recent studies have reported that meal timing may play an important role in weight regulation, however it is unknown whether the timing of meals is related to the amount of weight loss. This study aimed to examine the relationship between indices of meal timing and weight loss during weight loss intervention in adults. A 12-week weight loss support program was conducted for 97 adults (age: 47.6 ± 8.3 years, BMI: 25.4 ± 3.7 kg/m^2^). After the program, body weight decreased by −3.0 ± 2.7%. Only the start of the eating window was positively correlated with the weight change rate in both sexes (men: *r* = 0.321, *p* = 0.022; women: *r* = 0.360, *p* = 0.014). The participants were divided into two groups based on the start of the eating window as follows: the early group (6:48 ± 0:21 AM) and the late group (8:11 ± 1:05 AM). The weight loss rate in the early group was significantly higher (−3.8 ± 2.7%) than that in the late group (−2.2 ± 2.5%). The present results showed that the start of the early eating window was associated with weight loss and suggested paying attention to meal timing when doing weight loss.

## 1. Introduction

Traditional methods of body weight loss support for individuals with obesity and overweight have focused on the balance between energy expenditure and caloric intake. However, recent studies have shown that the circadian clock is associated with energy regulation at the behavioral, physiological, and molecular levels [1,2,3,4], emphasizing that meal timing itself may play an important role in body weight regulation [5,6,7].

A meta-analysis of observational studies examining the relationship between shift work and obesity has reported that shift workers and night shift workers have a higher incidence of obesity and lifestyle-related diseases [8], and nighttime eating has been shown to lead to weight gain [9,10]. For weight loss efficacy, some previous studies that examined the relationship between meal timing and weight loss effects have reported that early eaters have more weight loss than late eaters [11,12,13,14]. Moreover, recent meta-analyses of time-restricted feeding (TRF) and intermittent fasting have demonstrated weight loss effects [15,16]. These meta-analyses have also reported that intermittent fasting was more effective in reducing body weight than ad libitum feeding, although it was not clinically significant [15], and that the effect of TRF regimens in promoting weight loss was superior to that of approaches with unrestricted time in meal consumption [16]. Thus, previous studies have examined the relationship between each meal timing and weight loss, although time-of-day meal is associated with weight loss in free-living conditions has not been fully explored. Another recent study focused on time-of-day meal has reported that starting the eating window an hour later can negatively affect C-reactive protein, insulin, glucose, and high-density lipoprotein levels [17], although its association with weight loss has not been clarified.

Therefore, this study aimed to comprehensively evaluate the relationship between meal timing and the degree of weight loss and to explore the ideal meal timings and their factors.

## 2. Materials and Methods

### 2.1. Participants

The study participants were recruited from the portal sites of local government offices. Here, 60 adult men and 52 adult women who felt the need for weight loss requested participation. The recruitment criteria for the participants were those with a body mass index (BMI) of ≥20.0 kg/m^2^, who gained more weight than when they were 20 years old, and who had a smartphone. In case of participants with comorbidities, those who were not permitted by their family doctor to participate were excluded from the study. This research plan was approved by the Prefectural University of Kumamoto Bioethics Committee (approval numbers 30-30 and 01-20) and the Ethics Committee of the National Institutes of Biomedical Innovation, Health and Nutrition (approval number 122-01), and registered for clinical trials (UMIN33397). All participants were briefed on the purpose and content of the study prior to the intervention, and 59 men and 50 women agreed to participate in this study.

### 2.2. Study Design

**Figure 1:**
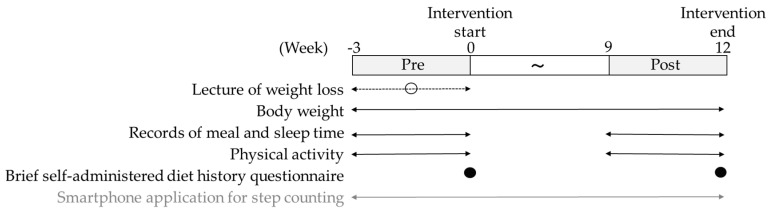
Intervention protocol. “Week” indicates the intervention period. The weight loss intervention period was weeks 0 to 12. The pre-measurement period was weeks −3 to 0, and the post-measurement period was weeks 9 to 12. The gray box indicates the pre-intervention and post-intervention periods. The double-headed arrow indicates that the measurement was performed during that period. The black circle indicates the point of the measurement. The white circle and dotted line indicate that the participants took the lecture of weight loss once during the period of dotted line. The gray letters and gray double-headed arrow indicate that the measurement was implemented in this intervention, although it was not relevant.

This study was a secondary analysis of “a study of the effect of using a smartphone application for step counting on weight loss”. The study was a randomized controlled trial of a 12-week weight loss support program in smartphone applications and non-smartphone applications (Figure 1). Weeks −3 to 0 (measurement before the intervention) were referred to as pre-intervention, and weeks 9–12 (measurement after the intervention) were referred to as post-intervention. After the research briefing, the participants attended a lecture on setting a body weight loss target of −5% from their current body weight, a dietary alternative based on −100 kcal/day and increasing physical activity (+1000 steps/day or more). Subsequently, the participants continued to measure their body weight and step counts in the application until the end of the intervention, and they provided updates on their weight loss and increased physical activity once a month by e-mail. In this intervention study, the weight loss rate was not significantly different between the application and non-application groups [18]. Additionally, no difference was observed in the weight change rate between the application and non-application groups for both men and women in this study. Therefore, there may be no difference in weight loss effects when using a smartphone application for step counting, and this study analyzed the two groups together as a single group.

### 2.3. Physical Measurement

Body weight was measured daily using a body composition analyzer (BC-308, Tanita Corporation, Tokyo, Japan), which can measure body weight in units of 50 g on an empty stomach after waking up, wearing light clothing as much as possible. Height was measured at the briefing session using a stadiometer (BW-306, Yamato Scale Co., Ltd., Hyogo, Japan), and the BMI at pre- and post-intervention was calculated. Data on body weight and measurement time were recorded in the device, and the average body weight for 3 weeks at pre- and post-intervention was adopted as a representative value.

### 2.4. Physical Activity

Physical activity was measured using a tri-axial accelerometer (Active style Pro HJA-750C; Omron Healthcare, Kyoto, Japan) [19] for 3 weeks at pre- and post-intervention. During the measurement period, the tri-axial accelerometer was set such that only the time could be displayed. From the data for 3 weeks, we adopted the days with ≥10 h of wear time per day [20,21,22], and calculated and evaluated the average value per day. Activity time by step count and intensity (≤1.5 METs (metabolic equivalents), 1.6–2.9 METs, and ≥3.0 METs) was evaluated as an index of physical activity. The number of days adopted for physical activity during the 3-week period was 19.5 days for pre-intervention and 18.5 days for post-intervention.

### 2.5. Circadian Timing of Daily Behaviors and Dietary Intake

Meal intake time, wake time, and bedtime were recorded in a self-administered format for 3 weeks at pre- and post-intervention, respectively. The midpoint of the eating window (the intermediate time from the start of the eating window to the end of the eating window) and fasting time (time elapsed between the end of the eating window to the start of the eating window of the next day) were calculated from the meal intake time. Sleep duration, midpoint of sleep, time elapsed between sleep offset and the start of the eating window, and time elapsed between the end of the eating window and sleep onset were calculated from meal intake time, wake time, and bedtime. The average value for each of the 3-week period was calculated and evaluated.

The Brief-type Self-administered Diet History Questionnaire (BDHQ) was used to assess dietary habits over the month before and after the intervention. The validity of the BDHQ has been confirmed [23,24,25]. Here, dietary energy intake and macronutrients were assessed using the BDHQ.

### 2.6. Statistical Analysis

Data represent the mean ± standard deviation (SD). All data were analyzed using SPSS Statistics 21 (IBM Japan, Ltd., Tokyo, Japan).

A Student’s paired *t*-test was used to compare body weight and energy intake at pre- and post-intervention. An analysis of covariance was performed to compare physical activity at pre- and post-intervention by adjusting wear time. Each of the effect sizes (ES) was calculated using G*power Version 3.1.9.7 software (Düsseldorf University, Düsseldorf, Germany). Pearson’s correlation analysis was used to examine factors related to meal timing and the weight change rate during the intervention.

A significant positive correlation was only observed between the start of the eating window and the weight change rate. From these results, the start of the eating window before intervention was divided into two groups by median using SPSS Statistics 21 to examine the relationship between their lifestyle and weight loss effects. This grouping was done for each sex so that the ratio of men to women participants would be equal. The group with the earlier start of the eating window was identified as the “early group”, and the group with the later start of the eating window was identified as the “late group”. A non-paired *t*-test was used to compare the early and late groups or the men and women participants. Regarding the comparison of the weight loss rate between the early and late groups, an analysis of covariance was performed by adjusting for age, sex, pre-BMI, and whether to use a smartphone application for step counting. Statistical significance was set at *p* < 0.05.

## 3. Results

Altogether, 109 individuals were screened for participation in the study. Finally, 51 men and 46 women, excluding those who dropped out (five men) and those who had missing or unanalyzable data (three men and four women), were included in the analysis. Table 1 shows the characteristics of the participants in the pre- and post-intervention results. Body weight and BMI were significantly reduced after the 12-week weight loss support program. The amount of weight loss in women was significantly lower than that in men (*p* = 0.010), although the rate of change in body weight was not different between men and women (*p* = 0.116). The energy intake was significantly lower at post-intervention than that at pre-intervention in men and women. The total and each intensity of physical activity time did not differ between pre- and post-intervention, although the step counts per hour of wear time increased post-intervention rather than pre-intervention.

**Figure 2:**
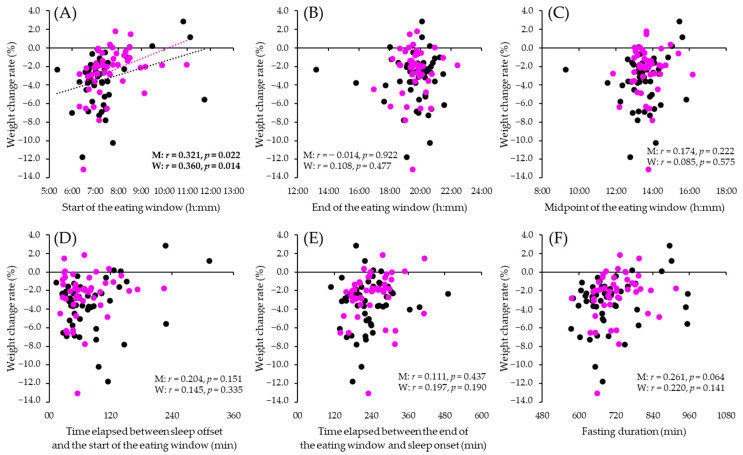
Correlation between meal timing and body weight loss. Scatter plots (black: men (*n* = 51); pink: women (*n* = 46)) show the correlations between weight change rate and the start of the eating window (**A**), the end of the eating window (**B**), the midpoint of the eating window (**C**), the time elapsed between sleep offset and the start of the eating window (**D**), the time elapsed between the end of the eating window and sleep onset (**E**), the fasting duration (**F**). M, men: W, women. The *p*-values were derived from the non-paired *t*-test, and “*r*” indicates the correlation coefficient by Pearson’s correlation analysis.

Regarding the relationship between meal timing at pre-intervention and weight change rate, only the start of the eating window was positively correlated with the weight change rate in both men and women (men: *r* = 0.321, *p* = 0.022; women: *r* = 0.360, *p* = 0.014) (Figure 2). No correlation was found between the other timing of meals (the end of the eating window, midpoint of the eating window, time elapsed between sleep offset and the start of the eating window, time elapsed between the end of the eating window and sleep onset and fasting time), and the weight change rate (Figure 2).

**Figure 3:**
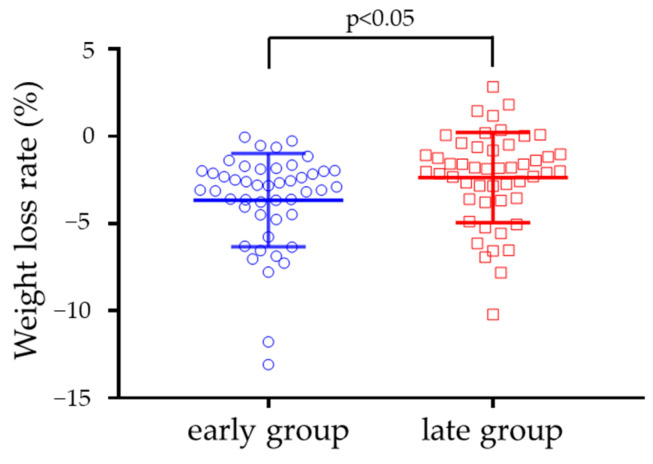
Weight loss rate (early group vs. late group). The long bars indicate the average, and the short bars indicate the standard deviation (blue: early group; red: late group). The blue circle indicates individual values of the early group, and the red square indicates individual values of the late group. The p-values were derived from the non-paired *t*-test. The weight loss rate in the early group was significantly greater than that in the late group when adjusted for sex, age, pre-body mass index, and use of a smartphone application for step counting by analysis of covariance (*p* = 0.033).

Based on these results, the median of the start of the eating window at pre-intervention was used to divide the men and women into the following two groups: the early group (25 men and 22 women) and the late group (26 men and 24 women). The start of the eating window was 6:48 ± 0:22 AM for the early group and 8:09 ± 1:05 AM for the late group in the pre-intervention (*p* < 0.001). The weight change rate was −3.8 ± 2.7% in the early group and −2.2 ± 2.5% in the late group (Figure 3). The weight loss rate in the early group was higher than that in the late group, even after adjusting for age, sex, pre-BMI, and use of a smartphone application for step counting (*p* < 0.05). 

The results of meal timing, physical activity, and dietary intake of the early and late groups are shown in Table 2. No difference was observed in sleep duration between the two groups, although wake time, bedtime, and midpoint of sleep in the early group were earlier than those in the late group. Furthermore, no difference was observed at the end of the eating window between the two groups, and the midpoint of the eating window in the early group was earlier than that in the late group. The time elapsed between sleep offset and fasting time was shorter in the early group than that in the late group. Regardless of pre- and post-intervention, the meal timing in both groups had almost the same statistical results (only the time elapsed between the end of the eating window and sleep onset in the post-intervention was later in the late group than that in the early group). Total energy intake did not differ between both groups at pre- and post-intervention. Physical activity was no difference in two groups between pre- and post- intervention; however, the activity time of both ≤1.5 METs and 1.6–2.9 METs in the late group were longer than those in the early group at both pre- and post-intervention. Step counts per hour of wear time increased post-intervention only in the early group. 

## 4. Discussion

The purpose of present study was to examine the relationship between meal timing and weight loss during the intervention in adults. The present results showed that the early eater group had significantly higher weight loss rate compared with the late eater group after adjusting sex, age and pre-BMI. These results indicate that meal timing may influence the weight loss success.

Consistent with the present results, some previous studies have shown that early eaters have more weight loss than late eaters [11,12,13]. In free-living weight loss intervention studies in Spain, Garaulet et al. [13] have reported that early lunch eaters lost more weight than late lunch eaters in obese people who followed a 20-week weight loss treatment program. A more recent study [11] has examined the eating time of 2116 people before a weight loss program, and it found that people with an early midpoint of the eating window (before 14:54, average ± SD: 14:27 ± 0:20) had an average 80 g higher weekly rate of weight loss than people with a late midpoint of the eating window (after 14:54, average ± SD: 15:25 ± 0:25). In a randomized crossover study in free-living conditions for 8 weeks, which implemented a strict set of meal times, the daytime eating schedule indicated better weight control than a delayed eating schedule [12]. The present study comprehensively examined the relationship between the related meal timing (time and interval) indices and weight loss rate and found that an earlier start of the eating window (early group: 6:48 ± 0:22 AM; late group: 8:09 ± 1:05 AM) likely results to a higher weight loss rate, which is similar to the results of previous studies. In contrast to these studies, although assessing the timing of food intake prior to weight loss intervention may not characterize low weight loss responders [26], their results have suggested that low weight loss responders were characterized by a lower proportion of energy intake until 9:00 AM. The difference in results might be due to differences in the methodology of the studies, such as differences in analysis method, definition of meal timing, and weight loss intervention. Interestingly, the first meal timing of the day was later between the pre- and post-intervention in the early group (06:48 AM vs. 07:00 AM, *p* < 0.001). This result might suggest that an earlier timing in the first meal of the day does not improve the weight loss effect, but rather that those who have a habit of eating at an earlier time are more likely to lose weight. Further research is needed to determine whether early mealtime habits or earlier timing in the intake of the first meal of the day is more effective for weight loss.

Energy balance, which is the gap between energy intake and expenditure, is the major determinant of body weight change, and a decrease in energy intake or an increase in energy expenditure is needed for weight loss. Although total energy intake, which was assessed using a validated self-administered diet history questionnaire, decreased in both groups during the weight loss program, the amount of decrease in energy intake post-intervention was not different between both groups in this study. Previous studies have reported that people who consumed the main meal early in the day or consume a high percentage during a morning meal had higher weight loss than those who consumed meals late [13,27,28,29], independent of energy intake, diet composition, and/or sleep duration [14] and partly consistent with the present results. The other factor is the influence of energy expenditure, such as physical activity, which is one of the factors related to circadian timing of daily behaviors. The present results showed that there was a significant negative correlation between weight change rate and post-step counts (*r* = −0.218, *p* = 0.033). Furthermore, the rate of total and absolute values for 1.6–2.9 METs in the early group were higher than those in the late group at pre- and post-intervention. Step counts per 60 min of wear time also increased at post-intervention compared with that at pre-intervention in the early group. Thus, physical activity may be one of the possible reasons for the more effective weight loss in the early group. A previous study has reported that a delay in meal timing is related to lower physical activity and %body fat [6]. Breakfast skippers had lower physical activity in a cross-sectional study [30], both in the later breakfast eating time intervention [31] and skipping breakfast intervention [32]. However, independent of physical activity levels, the relationship between later meal timing and higher BMI has been reported [13,33]. One of the reasons was that morning diet-induced thermogenesis (DIT) may partly result in weight loss success in early eaters. Early eating at a time earlier than in the afternoon and evening and then eating more in the morning or earlier might be effective in weight loss due to increased energy expenditure resulting from high DIT volume [34,35,36]. Therefore, a greater negative energy balance in the early group may have occurred than in the late group in this study. In the future, research on whether meal timing affects energy expenditure, such as physical activity and DIT, and the factors that lead to weight gain should be conducted in free living or experimental conditions. Additionally, research on the influence of eating time on energy intake and expenditure at the same time is also needed to understand of the relationship between meal-timing and the energy balance.

The longer the fasting duration, the greater the weight loss in TRF studies [37,38,39,40]. Here, the early group had a shorter fasting time (11:21 ± 1:05 AM) than the late group (12:21 ± 1:26 PM) despite a higher rate of weight loss, which is contrary to the results of previous studies. The conflicting results may be because the fasting duration in TRF studies that examined the effect on weight loss was 12–16 h, which is longer than that in this study and the decrease in the number of meals and total energy intake may have been one of the reasons for weight loss in TRF [38,39]. Here, the end of the eating window was similar in both groups, and the difference in the start of the eating window may have affected the difference in fasting time. The start of the eating window, regardless of a shorter fasting time, may be more effective for weight loss than a later start of the eating window to maintain a longer fasting time during weight loss in the real world condition. The participants in this study also had a last meal time of 19:27 ± 1:14 PM in the early group and 19:48 ± 1:05 PM in the late group, which is not too late. These results may be due to the fact that the participants were healthy adults with relatively well-organized lifestyles.

Our study is limited by the fact that these data were from a secondary analysis. The dietary assessment in this study only assessed the estimated dietary intake per day in the past month using the BDHQ, and not the dietary content at each meal. It is necessary to examine not only the timing of food intake, but also the content and amount of food intake. Moreover, this study’s results cannot be generalized because all the participants were employees of the local government and had relatively well-organized lifestyles. To clarify the relationship between meal timing and weight loss rate, it is necessary to further examine effects in the characteristics of the participants such as gender and lifestyle. Furthermore, among the data in this study, it should be noted that the data in the assessment of dietary intake and meal and sleep times are self-reported and need to be evaluated more objectively. Nevertheless, this study’s results suggest that the habit of starting an eating window at an earlier time in daily life may have a greater effect on weight loss, which is a useful finding for constructing an effective weight loss support program from the perspective of temporal nutrition. 

## 5. Conclusions

A greater weight loss effect was obtained in the population with an earlier start of the eating window in a 12-week weight loss support program. It will be necessary to consider the meal timing, along with the energy intake and the quality of meals, in order to obtain a high weight loss effect in the real world.

## Figures and Tables

**Table 1:** Characteristics of the participants (pre- vs. post-intervention).

	Total (*n* = 97)			Men (*n* = 51)			Women (*n* = 46)		
	Pre	Post	ES	Pre	Post	ES	Pre	Post	ES
Age (years)	47.2 ± 8.4	-	-	47.6 ± 8.7	-	-	46.9 ± 8.2	-	-
Height (cm)	165.5 ± 7.9	-	-	171.7 ± 3.8	-	-	158.6 ± 5.1	-	-
Body weight (kg)	70 ± 12.9	67.9 ± 12.8 **	0.163	77.6 ± 11.6	75 ± 11.8 **	0.222	61.5 ± 8.3	59.9 ± 8.3 **	0.193
BMI (kg/m^2^)	25.4 ± 3.7	24.7 ± 3.7 **	0.189	26.3 ± 3.7	25.4 ± 3.8 **	0.240	24.5 ± 3.4	23.8 ± 3.5 **	0.203
Body weight change (kg)	-	−2.1 ± 2	-	-	−2.6 ± 2.1	-	-	−1.6 ± 1.7	-
Body weight change rate (%)	-	−3.0 ± 2.7	-	-	−3.4 ± 2.7	-	-	−2.5 ± 2.6	-
**Dietary intake (BDHQ)**									
Total energy intake (kcal/day)	1900 ± 568	1732 ± 550 **	0.300	2122 ± 574	1910 ± 604 *	0.360	1654 ± 452	1533 ± 404 *	0.282
Total energy intake change (kcal/day)	-	−169 ± 442	-	-	−211 ± 553	-	-	−121 ± 269	-
Protein (%)	15.1 ± 2.6	16 ± 3.1 *	0.315	14.7 ± 2.7	15.6 ± 3.5 *	0.288	15.6 ± 2.6	16.4 ± 2.6 *	0.308
Fat (%)	28.1 ± 5.4	29.1 ± 6	0.175	26.1 ± 5.5	27.3 ± 6.5	0.199	30.3 ± 4.2	31.1 ± 4.6	0.182
Carbohydrate (%)	50.5 ± 7.3	48.5 ± 7.7 *	0.267	50.3 ± 8.1	47.8 ± 8.8	0.296	50.7 ± 6.4	49.3 ± 6.2	0.222
**Physical activity**									
Wearing time (min/day)	943 ± 105	917 ± 111 *	0.241	956.6 ± 116.8	908.6 ± 114.4 **	0.415	928.9 ± 88.3	927.1 ± 106.7	0.018
Step counts (steps/day)	7633 ± 3051	7876 ± 3234	0.045	8398 ± 3579	8823 ± 3729	0.055	6786 ± 2056	6827 ± 2172	0.011
≤1.5 METs (min/day)	590 ± 104	575 ± 94	0.013	620 ± 112	584 ± 93	0.013	557 ± 83	564 ± 95	0.055
1.6–2.9 METs (min/day)	295 ± 72	284 ± 74	0.022	276 ± 67	262 ± 68	0.007	317 ± 73	309 ± 74	0.055
3.0 METs ≤ (min/day)	58 ± 24	58 ± 24	0.022	61 ± 28	63 ± 28	0.045	54 ± 19	53 ± 19	0.032
≤1.5 METs (%)	62.3 ± 7.4	62.5 ± 7.4	0.027	64.4 ± 7	64.1 ± 6.6	0.044	60 ± 7.3	60.7 ± 7.9	0.092
1.6–2.9 METs (%)	31.5 ± 6.9	31.1 ± 7	0.058	29.1 ± 6.4	28.9 ± 6.3	0.031	34.1 ± 6.6	33.5 ± 7.1	0.088
3.0 METs ≤ (%)	6.2 ± 2.7	6.4 ± 2.7	0.074	6.5 ± 3.1	7 ± 3.2	0.159	5.9 ± 2.1	5.8 ± 1.9	0.050
Step counts (steps/h wear time)	492 ± 206	520 ± 221 *	0.131	536 ± 242	589 ± 260 *	0.211	443 ± 145	443 ± 134	0.000
≤1.5 METs (min/h wear time)	37.5 ± 4.5	37.6 ± 4.5	0.022	38.8 ± 4.2	38.6 ± 4	0.049	36 ± 4.4	36.6 ± 4.7	0.132
1.6–2.9 METs (min/h wear time)	18.8 ± 4.2	18.5 ± 4.2	0.071	17.4 ± 3.9	17.2 ± 3.8	0.052	20.4 ± 4	20 ± 4.2	0.098
3.0 METs ≤ (min/h wear time)	3.7 ± 1.6	3.8 ± 1.6	0.063	3.9 ± 1.9	4.2 ± 1.9	0.158	3.5 ± 1.3	3.4 ± 1.1	0.083

Mean ± SD. * *p* < 0.05, ** *p* < 0.001. Letters in parentheses indicate units. ES indicates the effect size. Bold face indicates statistical differences with *p* < 0.05. A paired *t*-test was used for comparison of pre- vs. post-intervention. For the results of physical activity time (min/day), an analysis of covariance adjusted for wearing time was used in comparing physical activity. BMI, Body mass index; BDHQ, Brief-type Self-administered Diet History Questionnaire; METs, metabolic equivalents; h, hour.

**Table 2:** Characteristics of the participants (early group vs. late group).

	Early Group (*n* = 47, Men: 25/Women: 22)			Late Group *(n* = 50, Men: 26/Women: 24)			Early Group vs. Late Group	
							Pre	Post
	Pre	Post	ES	Pre	Post	ES	ES	ES
Start of eating window (h:mm)	6:48 ± 0:22	7:00 ± 0:30 *	0.429	8:09 ± 1:05 ††	8:19 ± 1:27 ††	0.127	1.649	1.214
Age (years)	48.8 ± 6.6	-	-	45.8 ± 9.7	-	-	0.362	-
Height (cm)	165.4 ± 7.1	-	-	165.5 ± 8.7	-	-	0.013	-
Body weight (kg)	69.3 ± 12.1	66.7 ± 11.6 **	0.219	70.6 ± 13.7	68.9 ± 13.8 **	0.124	0.101	0.173
BMI (kg/m^2^)	25.2 ± 3.6	24.3 ± 3.4 **	0.257	25.6 ± 3.8	25.0 ± 4.0 **	0.154	0.108	0.189
Body weight change (kg)	-	−2.6 ± 2.0	-	-	−1.6 ± 1.9 †	-	-	0.513
Body weight change (%)	-	−3.7 ± 2.7	-	-	−2.4 ± 2.6 †	-	-	0.490
**Meal and sleep timing**								
Wake time (h:mm)	5:54 ± 0:33	6:05 ± 0:36 **	0.306	6:40 ± 0:42 ††	6:46 ± 0:50 ††	0.124	1.212	0.937
Bedtime (h:mm)	23:17 ± 0:55	23:15 ± 0:56	0.030	23:49 ± 1:00 †	23:57 ± 0:55 ††	0.135	0.549	0.744
Sleep duration (min)	396 ± 50	409 ± 49 *	0.263	410 ± 50	408 ± 43	0.053	0.281	0.031
Midpoint of sleep (h:mm)	26:35 ± 0:38	26:40 ± 0:40	0.121	27:15 ± 0:46 ††	27:22 ± 0:48 * ††	0.143	0.919	0.927
End of eating window (h:mm)	19:27 ± 1:14	19:22 ± 1:00	0.075	19:48 ± 1:05	19:37 ± 1:05 *	0.168	0.306	0.247
Midpoint of eating window (h:mm)	13:23 ± 0:50	13:11 ± 0:36 *	0.276	13:47 ± 0:55 †	13:59 ± 1:01 ††	0.194	0.467	0.944
Time elapsed between sleep offset and start of eating window (min)	57 ± 25	56 ± 26	0.039	91 ± 62 †	93 ± 78 †	0.028	0.719	0.636
Time elapsed between end of eating window and sleep onset (min)	231 ± 71	231 ± 61	0.000	240 ± 59	259 ± 60 * †	0.319	0.138	0.463
Fasting duration (min)	681 ± 65	696 ± 60*	0.240	741 ± 86 ††	759 ± 92 * ††	0.202	0.787	0.811
**Dietary intake**								
Total energy intake (kcal/day)	1818 ± 542	1622 ± 390 *	0.415	1977 ± 586	1834 ± 654 *	0.230	0.282	0.394
Total energy intake change (kcal/day)	-	−196 ± 389	-	-	−143 ± 489	-	-	0.120
Protein (%)	15.4 ± 2.4	16.1 ± 3.3	0.243	14.9 ± 2.9	15.9 ± 3.0 *	0.339	0.188	0.063
Fat (%)	28.2 ± 5.4	28.0 ± 6.2	0.034	28.0 ± 5.4	30.2 ± 5.6 *	0.400	0.037	0.372
Carbohydrate (%)	50.5 ± 7.6	49.5 ± 8.5	0.124	50.5 ± 7.1	47.6 ± 6.8 *	0.417	0.000	0.128
**Physical activity**								
Wearing time (min/day)	987 ± 105	955 ± 112 *	0.295	903 ± 88 ††	882 ± 98 * †	0.225	0.867	0.200
Step counts (steps/day)	7607 ± 2749	8233 ± 2873	0.132	7658 ± 3337	7541 ± 3536	0.032	0.000	0.084
≤1.5 METs (min/day)	603 ± 116	579 ± 101	0.007	578 ± 91 †	570 ± 88 †	0.063	0.279	0.291
1.6–2.9 METs (min/day)	324 ± 73	313 ± 69	0.032	269 ± 61 †	257 ± 69 †	0.045	0.281	0.261
3.0 METs ≤ (min/day)	59 ± 24	62 ± 23	0.095	56 ± 25	54 ± 26	0.045	0.045	0.115
≤1.5 METs (%)	60.8 ± 8.2	60.4 ± 7.2	0.052	63.7 ± 6.4	64.4 ± 7.1 †	0.104	0.394	0.559
1.6–2.9 METs (%)	33.1 ± 7.3	33.0 ± 6.4	0.015	29.9 ± 6.3 †	29.3 ± 7.1 †	0.089	0.469	1.248
3.0 METs ≤ (%)	6.1 ± 2.5	6.6 ± 2.4	0.204	6.4 ± 2.9	6.3 ± 3.1	0.033	0.111	0.108
Step counts (steps/h wear time)	467 ± 173	520 ± 188 *	0.293	515 ± 232	520 ± 250	0.021	0.235	0.000
≤1.5 METs (min/h wear time)	36.6 ± 4.9	36.4 ± 4.4	0.043	38.3 ± 3.9	38.8 ± 4.3 †	0.122	0.384	0.552
1.6–2.9 METs (min/h wear time)	19.8 ± 4.4	19.7 ± 3.9	0.024	17.9 ± 3.8 †	17.4 ± 4.2 †	0.125	0.462	0.568
3.0 METs ≤ (min/h wear time)	3.6 ± 1.5	3.9 ± 1.4	0.207	3.8 ± 1.7	3.7 ± 1.8	0.057	0.125	0.124

Mean ± SD. * *p* < 0.05, ** *p* < 0.001 (pre vs. post). † *p* < 0.05, †† *p* < 0.001 (early group vs. late group). Letters in parentheses indicate units. ES indicates the effect size. Bold face indicates statistical differences with *p* < 0.05. A paired *t*-test was used for pre- vs. post-intervention comparison, and a non-paired *t*-test was used for comparison of the early group vs. the late group. For the results of physical activity time (min/day), an analysis of covariance adjusted for wearing time was used in comparing physical activity. BMI, Body mass index; BDHQ, Brief-type Self-administered Diet History Questionnaire; METs, metabolic equivalents; h, hour.

## Data Availability

The datasets generated and/or analyzed during the current study are available from the corresponding author on reasonable request, pending ethics approval.
